# Editorial: Addiction and Attachment

**DOI:** 10.3389/fpsyt.2020.612044

**Published:** 2020-11-27

**Authors:** Andrew J. Lewis, Human F. Unterrainer, Megan Galbally, Andreas Schindler

**Affiliations:** ^1^Discipline of Psychology, Murdoch University, Perth, WA, Australia; ^2^Center for Integrative Addiction Research, Grüner Kreis Society, Vienna, Austria; ^3^University Clinic for Psychiatry and Psychotherapeutic Medicine, Medical University Graz, Graz, Austria; ^4^Department of Religious Studies, University of Vienna, Vienna, Austria; ^5^King Edward Memorial Hospital, Perth, WA, Australia; ^6^School of Medicine, Notre Dame University, Perth, WA, Australia; ^7^University Medical Center Eppendorf, Hamburg, Germany

**Keywords:** attachment, addiction, substance use disorder, trauma, evolution

In the postscript to “Attachment Across the Life Cycle,” John Bowlby wrote: “Once we postulate the presence within the organism of an attachment behavioral system regarded as the product of evolution and as having protection as its biological function, many of the puzzles that have perplexed students of human relationships are found to be soluble” (1). This statement displays Bowlby's theoretical integration of evolutionary, functional, and behavioral levels of analysis, thereby giving attachment theory the ability to investigate a wide range of social and emotional relationships at both psychological and biological levels.

There is a complex interaction between a person's attachment history, the quality of early experiences, and their propensity to addictive behaviors. Clinicians and therapists working in the addiction field address this reality on a daily basis, but the research that brings precision, systematization, and the capacity to test these assumptions is only beginning to gather pace. Connecting research in the two areas of attachment and addiction can be beneficial to each; the neurobiology of basic motivational systems of social affiliation might help us understand behavioral patterns and motivations of addiction, while the biology of addiction might help us identify the evolved systems underlying attachment.

In May 2018, in the grounds of the *Schloß Schönbrunn* in Vienna nearly 400 delegates gathered to discuss the many facets of the relationship between attachment and addiction. Following the success of the “*Sucht und Bindung” [Addiction and Attachment]* conference—graciously hosted by the *Grüner Kreis* Society—we put out a call for papers for a Frontiers Research Topic. We were delighted to receive 22 high quality papers providing both original studies, reviews of the latest findings, theoretically oriented discussions, and applications to clinical treatments.

## Section 1: Treatment Approaches and Models of Addiction and Attachment

As an integrative psychological and biological theory of social affiliation, attachment research includes neurobiological, neuroimaging, and endocrine studies of the biological correlates of social affiliation and bonding across many mammalian species. Schindler notes a substantial growth in the literature on addiction and attachment over the last decade. His review of 34 cross-sectional studies, three longitudinal studies, and a recent meta-analysis suggests that there is consistent evidence of an association between insecure attachment and substance abuse both cross-sectionally and in longitudinal studies. There is also evidence in studies of a bidirectional relationship which accounts for how substance use also predicts a deterioration in attachment relationships. The specific patterns of the attachment involved remains less clear, but several studies suggest that fearful–avoidant attachment may be more frequent in heroin addicts, while alcohol abuse tends to display more heterogeneous patterns. Hiebler-Ragger and Unterrainer also contribute a review of the theories on the development and treatment of Substance Use Disorders (SUD). Their review covers the interaction between addiction and attachment also from a neuroscientific perspective, suggesting that changes in brain white matter integrity may be involved in the emotional dysregulation associated with substance use.

Attachment ideas can be extended beyond dyadic relations to enhance our understanding of the inter-subjective dynamics of family functioning and community-based interventions. In this regard Lewis presents a theoretical overview and description of the treatment approach for an attachment based intervention used to treat adolescent substance use and mental health. The intervention focuses on attachment dynamics across whole families and has been successfully evaluated over several trials. On a similar theme, Heerde et al. leads a paper using comparative data from Australia and the USA on family environmental factors as predictors of substance use in young adults. Finally, De Leon and Unterrainer present an overview of many decades of work pioneering the therapeutic community approach to the treatment of addictions, drawing in links between a therapeutic community and the attachment needs of its members.

## Section 2: Evolutionary Hypotheses and Neurobiological Models

Another major theme to emerge from the contributions shows the value in attachment theory as a psychobiological model grounded in developmental and evolutionary hypotheses as a function of attachment behavior. This produces one of the major hypotheses of attachment research—that such functions must be derived from evolved mechanisms that are inherent in neurobiological systems and emerge at critical periods of ontogeny. Strathearn et al. leads a review that addresses the developmental pathway through which compromised early experience—which includes insecure and disorganized attachment but also early abuse, neglect, or trauma—influences later susceptibility to addictions later in life. This paper provided a detailed account of the neurobiological pathways associated with this developmental pathway. Currently, studies focus on the oxytocin system of social affiliation, the role of dopamine, and opioid reward systems and alterations in the corticoid stress response system. Mosri presents a novel model of addiction drawing on neuro-psychoanalysis and Pankseep's model of separation distress as a compromise of opioid regulation. This paper integrates neurobiological and subjective data which conceptualizes addiction as a latent type of depression.

We then have three empirical studies of the neurobiology of attachment and addiction. First a study lead by Unterrainer et al. reporting brain structure modifications in Poly-Drug Users (PUD), specifically cortical thickness and White Matter Impairments, second Fuchshuber, Tatzer et al. lead a study reporting differences in oxytocin reactivity in an experimental study of SUD patients compared to controls in which the Adult Attachment Projective was used as an attachment stimulus, and third Torres examines a neuro-evolutionary model of social bonding finding that methadone use predicts reduced Attachment Anxiety, Comfort With Closeness, and Proximity Maintenance.

## Section 3: Trauma and Attachment

Four papers addressed the relationship between attachment, trauma, and addiction. Lahousen et al. present a comprehensive overview of the evolutionary foundations of attachment theory and consider how this is compromised by attachment trauma. This paper draws in links between trauma and dissociation to account for the vulnerability in emotional dysregulation which leads to addiction. Meulewaeter et al. leads a paper presenting the findings of a qualitative study of substance using mothers to examine the impact of trauma and its role as a precipitant of substance use. Lotzin et al. present important data from a large sample of treatment-seeking women with SUD and comorbid posttraumatic stress disorders. Using the Childhood Trauma Questionnaire, they distinguish four different childhood trauma profiles showing that those with more severe levels of childhood trauma show an earlier age at initiation and escalation of substance use. To highlight the growth in the literature that examines trauma and addiction, Rinker provides a book review of a collection edited by Richard Gill: “Addictions From an Attachment Perspective: Do Broken Bonds and Early Trauma Lead to Addictive Behavior?”

## Section 4: Attachment, Emotion Regulation, and Personality Functioning

This section features a total of six contributions, which address the relationship between addiction, deficient emotional and personality dysfunction, seen through the lens of attachment theory. In the first, an empirical study based on a population sample, conducted by Fuchshuber et al. (b), showed that attachment styles, personality organization, and emotional functioning after traumatic experiences in childhood are interconnected. Based on their results the authors conclude that SUD treatment might be improved by focusing on facilitating the development of more secure attachment patterns and improved personality functioning. In line with these findings, a study lead by Ramirez-Castillo et al., found a significantly lower amount of Frustration Tolerance as being related to disorganized attachment in SUD-patients. This suggests that Frustration Tolerance might also be seen as an important factor to consider in addiction treatment programs. Along similar lines, Yang et al. present findings from a cross-sectional assessment of 298 SUD patients which suggests that an increase in self-control, resilience, and self-esteem contributes to improved self-efficacy in SUD-patients.

Furthermore, in another study conducted by Fuchshuber et al. (a) data from a community sample is used to examine the predictive value of primary emotions for psychopathological symptoms by means of a Multi-group Path Analysis. In line with the hypothesis, primary emotions were observed to be a substantial predictor or even promotor for the development of psychopathology. Based on these findings it is concluded that primary emotion functioning could also be a valuable target in mental health care. Furthermore, in a study done by Vismara et al. the quality of attachment in SUD patients is investigated in order to identify the role of attachment security in choosing a suitable treatment facility. In this paper substantial differences in attachment styles between SUD out- and inpatients are reported. Consequently, the authors conclude that considering the variability of attachment patterns in SUD patients, could also contribute to improved interventions. Lastly, Jauk and Dieterich review the literature on the relationship between the Dark Triad of personality (Narcissism, Machiavellianism, and Psychopathy) and addictive behaviors, both substance-related and non-substance-related. In a similar way to the other papers in this section, they conclude that considering such parameters of personality functioning is of real importance for a better understanding and treatment of addictive disorders.

## Section 5: New Modes of Addiction to Social Media

Finally the Research Topic presents two papers describing new forms of addiction related to the excessive use of smartphones and the internet lead by Eichenberg et al. and Jung et al., respectively. In the first study, attachment patterns predicted problematic smartphone usage and specifically lower attachment security. In the second study, participants with larger social networks and higher scores in the received social support showed the lowest rates of Internet Use Disorder (IUD).

In addressing the topic of *Addiction and Attachment*, this Research Topic has identified five broad research areas. The reviews presented show strong progress in the field, with a growing application of attachment models and measures to the challenge of addiction. While much of the empirical research presented is exploratory this Research Topic provides clear direction for future research endeavors and confirms the importance and relevance of attachment theory in understanding addiction.

## Author Contributions

AL and HU wrote the first draft of the manuscript. MG and AS provided critical revision of the manuscript and important intellectual contributions. All authors read and approved the submitted version.

## Conflict of Interest

The authors declare that the research was conducted in the absence of any commercial or financial relationships that could be construed as a potential conflict of interest.
